# CT-gesteuerte Schmerztherapie des spezifischen Rückenschmerzes: Bandscheibenschädigung vs. ossäre Degeneration

**DOI:** 10.1007/s00117-021-00850-2

**Published:** 2021-05-12

**Authors:** Christoph. A. Stueckle, Sarah Talarczyk, Kerstin F. Stueckle, Patrick Haage

**Affiliations:** 1 Fakultät für Gesundheit, Universität Witten/Herdecke, Witten, Deutschland; 2 Zentrum für Radiologie, Helios Universitätsklinikum Wuppertal, Wuppertal, Deutschland; 3 Deutsche Rentenversicherung Knappschaft Bahn-See, Bochum, Deutschland; 4 Institut für Schnittbildgebung Dr. Amirfallah, Europaplatz 11, 44141 Dortmund, Deutschland; 5 MVZ Prof. Uhlenbrock, Dortmund, Deutschland

**Keywords:** Rückenschmerz, Facettentherapie, Spondylarthropathie, Intervention, Computertomographie, Back pain, Facet therapy, Spondylarthropathy, Intervention, Computed tomography

## Abstract

**Hintergrund:**

Rückenschmerz ist häufig und führt den Patienten sowohl zur Diagnostik als auch in bestimmten Fällen für eine Therapie zum Radiologen.

**Fragestellung:**

Die vorliegende Untersuchung vergleicht die schmerzreduzierende Wirkung der mikroinvasiven Computertomographie(CT)-gesteuerten Schmerztherapie bei diskogenem und spondylarthrotisch bedingtem spezifischem Rückenschmerz.

**Material und Methode:**

Über einen Zeitraum von 3,3 Jahren wurden 239 Patienten in die Untersuchung eingeschlossen, bei denen 686 CT-gesteuerte periradikuläre Therapien (PRT) und 264 CT-gesteuerte Facettengelenktherapien (FAC) durchgeführt und beurteilt wurden. Bei allen Patienten wurde vor der Intervention, im Verlauf und am Ende der Schmerzscore mittels visueller analoger Schmerzskala (VAS) bestimmt. Abschließend wurde der Behandlungserfolg in Abhängigkeit von der durchgeführten Behandlungsart und den morphologisch vorliegenden Veränderungen korreliert.

**Ergebnisse:**

In beiden Gruppen zeigte sich unter der Behandlung eine gute Beschwerdebesserung (74 % bei PRT-Patienten und 60 % bei FAC-Patienten). Die Patienten, bei denen eine PRT durchgeführt wurde, zeigten durchschnittlich eine Verbesserung des Schmerzscores von 3,1, bei Patienten mit durchgeführter FAC von 2,1. Die Wirksamkeit der FAC zeigte eine Abhängigkeit der Wirksamkeit vom Grad der vorhandenen degenerativen Veränderungen. Je ausgeprägter die nachgewiesene Degeneration im behandelten Segment war, desto mehr Interventionen waren für ein gutes Therapieansprechen notwendig.

**Schlussfolgerung:**

Die CT-gesteuerte PRT und FAC führen beide zu einer guten Reduktion der Beschwerdesymptomatik. Im Vergleich erzielte die PRT eine signifikant höhere Schmerzreduktion als die FAC.

## Abstract (en)

**Background:**

Back pain is common and leads the patient to the radiologist both for diagnosis and in specific cases for therapy.

**Objectives:**

The current study compares the pain-reducing effect of microinvasive computed tomography (CT)-guided pain therapy for specific back pain caused by herniated discs or spondylarthrosis.

**Materials and methods:**

Over a period of 3.3 years, a total of 239 patients were included, in whom 686 CT-guided periradicular therapies (PRT) and 264 CT-guided facet joint therapies (FAC) were performed. In all patients, the pain score was determined using a visual analog pain scale (VAS) before the intervention, during the course of treatment and at the end of treatment. Finally, treatment success was correlated to the type of treatment performed and to the morphological changes present.

**Results:**

Both groups showed good improvement of pain under treatment (74% of the PRT patients and 60% of the FAC patients). Patients who underwent PRT showed an average improvement in pain score of 3.1, while those who underwent FAC showed an average improvement of 2.1. The efficacy of FAC was dependent on the degree of degenerative changes present. The more extensive the proven degeneration was in the treated segment, the more interventions were necessary for a good treatment response.

**Conclusion:**

CT-guided PRT and CT-guided FAC both lead to a good reduction of pain symptoms. In comparison, PRT achieves significantly higher pain reduction than FAC.

Beschwerden im Bereich des Rückens, die zum Aufsuchen des Orthopäden oder des Hausarztes und dann zur Konsultation des Radiologen führen, sind häufig. Da das Symptom Rückenschmerz multifaktoriell bedingt ist, empfehlen die Leitlinien erst nach 6 Wochen oder bei Vorliegen sog. „red flags“ eine Bildgebung [8]. Neben der entsprechenden Schnittbildgebung zur Darstellung der Schmerzursache fällt dem Radiologen auch zunehmend die Aufgabe der (Mit‑)Behandlung des Rückenschmerzes in Form von CT-gesteuerten Schmerzinterventionen zu.

## Hintergrund

Der radiologischen Therapie zugänglich ist der spezifische Rückenschmerz, bei dem sich eine pathoanatomische Ursache des Schmerzes diagnostizieren lässt [2, 13]. Zur Diagnostik des Rückenschmerzes und zum Ausschluss von nichtspezifischem Rückenschmerz stehen die Magnetresonanztomographie (MRT) und die Computertomographie (CT) zur Verfügung, mit welcher die pathoanatomischen Ursachen des Rückenschmerzes zuverlässig detektiert werden können [9, 18]. Hierbei stellt zunächst die MRT die Methode der Wahl dar. Bei mangelnder Verfügbarkeit der MRT oder vorliegender Kontraindikation ist die CT mit Anfertigung multiplanerer Rekonstruktionen eine Alternative [8, 35]. Für das konventionelle Röntgen liegt lediglich eine Empfehlung zum Nachweis von Deformierungen der Wirbelsäule vor [8].

In Deutschland werden mehrere Therapieansätze beim Rückenschmerz und insbesondere beim chronischen und rezidivierenden Rückenschmerz genutzt, die zum Teil aufeinander aufbauen. Eingesetzt werden die CT-gesteuerte Schmerztherapie, physikalische und manuelle Therapie, Rückenschule und Bewegungstherapie, Pharmakotherapie, Verhaltenstherapie, Krankheitsaufklärung sowie operative Maßnahmen. In den letzten Jahren zeigt sich ein Trend hin zur multimodalen Schmerztherapie auch unter Einschluss der radiologischen Schmerztherapie, welche insbesondere bei der Entwicklung chronischer Rückenschmerzen Anwendung findet [5, 32].

Ein häufiger lokaler pharmakotherapeutischer Ansatz bei spezifischen Rückenschmerzen ist die CT-gesteuerte Schmerztherapie mit lokaler Steroidinjektion, wobei zwischen der periradikulären und epiduralen Infiltration sowie der Facettentherapie unterschieden wird [16]. Die periradikuläre bzw. epidurale Therapie findet Anwendung bei der Radikulopathie, d. h. ein radikulärer Schmerz, hervorgerufen durch eine Nervenwurzelirritation im Rahmen mechanischer Kompression und resultierender Inflammation mit entsprechendem Dermatombezug [11, 16]. Die häufigsten Ursachen für eine Radikulopathie bzw. mechanische Nervenwurzelkompression im Lendenwirbelsäulenbereich sind, neben Bandscheibenherniationen, osteogene degenerative Veränderungen: hypertrophe Spondylarthrose, Spondylolisthesis und Hypertrophie der Ligamenta flava [25], wobei als häufigste Ursache die Bandscheibenschädigung berichtet wird [1, 4]. Die Facetteninfiltration hingegen findet Anwendung beim Facettensyndrom, einem Schmerz im Bereich der Facettengelenke, hervorgerufen durch segmentale Instabilität, Synovitis oder degenerative Arthritis [24]. Steroidinjektionen – sowohl periradikulär bzw. epidural im Rahmen einer Radikulopathie, als auch unmittelbar angrenzend an die Facettengelenke im Rahmen eines Facettensyndroms – werden zur Reduktion der Inflammation und letztlich zur Schmerzreduktion eingesetzt, was wiederum den Verbrauch oraler Schmerzmedikamente und die Notwendigkeit einer Operation vermindern kann [17]. Da sich in mehreren Studien keine eindeutige Korrelation zwischen dem Bildbefund und der berichteten Schmerzsymptomatik zeigte, ist eine präzise Patientenselektion, in welcher der bildmorphologische Befund die präsentierte Klinik erklärt, essenziell für die Therapieplanung und den Therapieerfolg einer CT-gesteuerten Schmerztherapie [36].

## Zielsetzung

Die vorliegende Untersuchung vergleicht die Wirksamkeit der mikroinvasiven CT-gesteuerten Schmerztherapie bei diskogenem und spondylarthrotisch bedingtem spezifischem Rückenschmerz.

## Material und Methode

Die prospektive Untersuchung erfolgte im Zeitraum von 11/2016 bis 03/2020 nach positivem Votum der Ethikkommission der Universität Witten/Herdecke. Alle eingeschlossenen Patienten litten an spezifischen und zur Klinik passenden zervikalen oder lumbalen Rückenschmerzen mit MR-morphologisch nachgewiesener diskogener oder spondylarthrotischer Schmerzursache. Patienten mit neoplastischen und traumatischen Pathologien sowie Voroperationen an der Wirbelsäule wurden ausgeschlossen, ebenso Patienten mit Verdacht auf oder nachgewiesener Spondylodiskitis. Ebenfalls ausgeschlossen wurden Patienten, die zum Zeitpunkt der Aufklärung oder Intervention Schmerzmedikamente einnahmen oder eine zusätzliche physiotherapeutische oder psychologische Behandlung bekamen. Eingeschlossene Patienten mit diskogener Schmerzursache/diskogener Nervenwurzelaffektion erhielten im Verlauf eine periradikuläre Therapie, Patienten mit spondylarthrotischer Schmerzursache eine Facetteninfiltration. Die Indikationsbesprechung erfolgte jeweils interdisziplinär zwischen behandelndem Radiologen, Orthopäden, Schmerztherapeuten und Neurologen. Bei Einschluss der Patienten in die Untersuchung wurden die Klinik, deren bildmorphologisch diagnostizierte Ursache sowie die zur Verfügung stehenden Behandlungsmethoden einschließlich des Risiko- und Eignungsprofil des Patienten berücksichtigt.

Eine diskogene Nervenwurzelaffektion wurde immer dann diagnostiziert, wenn in der MRT eine Bandscheibenherniation mit nachweisbarem Kontakt/nachweisbarer Kompression zwischen Bandscheibengewebe und Nerv vorlag. Der Grad der Spondylarthrose wurde nach Kellgren-Lawrence in modifizierter Form eingeteilt:

- *Grad 1* im Sinne einer bildmorphologisch abgrenzbaren Spondylarthrose ohne signifikante Hypertrophie,
- *Grad 2* im Sinne eine Spondylarthrose mit Hypertrophie aber ohne Destruktion der Gelenkpartner,
- *Grad 3* im Sinne einer stark fortgeschrittenen Spondylarthrose mit zusätzlich abgrenzbarer Destruktion der Gelenkpartner [21, 22].

Vor der geplanten Intervention und vor Einschluss in die Untersuchung erfolgten eine ausführliche Schmerzanamnese, Untersuchung und Interventionsaufklärung sowie – falls die Patienten ihr Interesse bekundeten – eine Information der Patienten über die Studie. Alle hier eingeschlossenen Patienten erklärten sich sowohl mit der Intervention als auch mit der Datenerhebung im Rahmen der Studie einverstanden.

An den Tagen der CT-gesteuerten periradikulären Therapie/der CT-gesteuerten Facettengelenktherapie wurden die Patienten vor dem Eingriff hinsichtlich ihrer aktuellen Schmerzen befragt. Die Patienten wurden gebeten, ihren Schmerzscore auf einer visuellen Analogskala (VAS) einzuordnen. Die verwendete VAS wies eine Skalierung von 0 bis 10 auf (0 bedeutet „kein Schmerz“, 10 „der schlimmste vorstellbare Schmerz“). Als Beschwerdebesserung wurde eine Verbesserung des Schmerzscores um 25 % oder mehr unter Therapie definiert.

Insgesamt 5 Kolleg*innen mit jeweils mehr als 500 eigenständig durchgeführten Schmerzinterventionen führten an 2 Zentren in zufälliger Auswahl die Interventionen durch. Die eingeschlossenen Untersuchungen wurden jeweils von einem sehr erfahrenen Kollegen hinsichtlich ihrer Qualität und Durchführung beurteilt.

**Figure 1:**
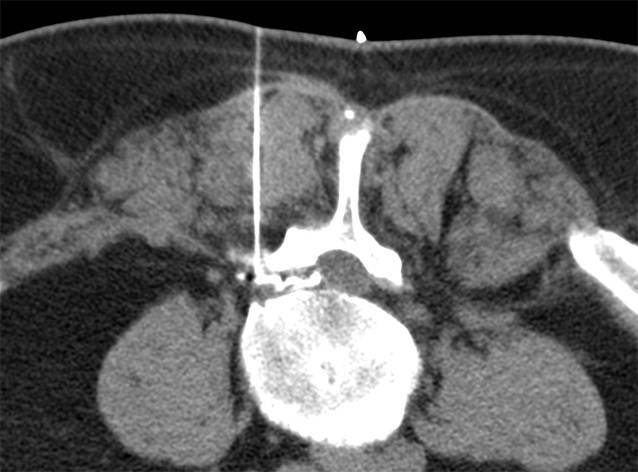

Alle Interventionen wurden in Bauchlage durchgeführt. Die Planung der Untersuchung erfolgte jeweils an einem angepassten Planungsscout auf der zuvor bekannten Interventionshöhe. Anschließend wurde die Interventionsplanung in der entsprechenden CT-Schicht durchgeführt. Nach sorgfältiger lokaler Desinfektion, steriler Vorbereitung und Markierung der Punktionsstelle wurde unter CT-Kontrolle in Low-dose-Technik unter Einsatz von CareDose die Intervention durchgeführt (Tab. 1). Nach Maßgabe des behandelnden Radiologen erfolgte die Überprüfung der Nadelposition während der Intervention, und die Verteilung des Medikamentengemisches wurde dokumentiert (Abb. 1 und 2). Die Interventionen wurden entweder an einem Siemens Emotion 6, an einem Siemens Somatom Definition 64 oder an einem Siemens Definition AS 64 durchgeführt.

**Table 1:** 

	Siemens Somatom Definition 64	Siemens Definition AS 64	Siemens Emotion 6
KV	120	120	110
mAs	48–60	48–60	48–60
Kollimation	12 × 1,2 mm	12 × 1,2 mm	6 × 3 mm
Dosisanpassung	CareDose 4D	CareDose 4D + Care KV	Care Dose

Übersicht der eingesetzten Scanner und der benutzten Scan-Parameter bzw. Programme. Unterschiedliche werksseitige Dosismodulation aufgrund verschiedener Software Entwicklungsstufen

**Figure 2:**
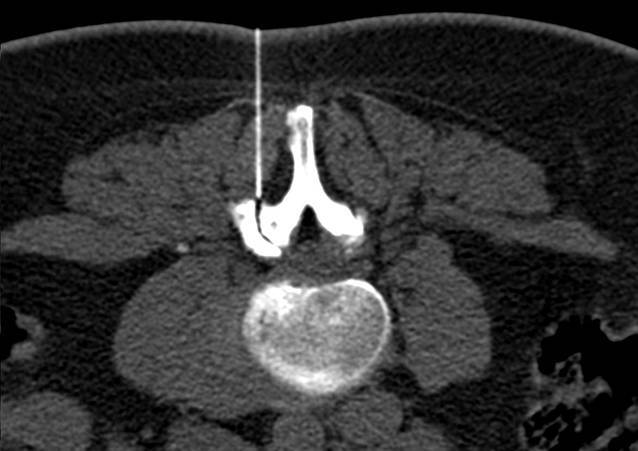

In Abhängigkeit von der körperlichen Fülle des Patienten wurde eine 22G-Nadel mit einer Länge von 90 mm oder 120 mm verwendet (Becton Dickinson SA, S. Agustin del Gualdix, Spain). Als Lokalanästhetikum wurde 2 %iges Meaverin eingesetzt (Meaverin 2 %, Puren Pharma, Munich, Germany). Als Kontrastmittel zur Dokumentation der Verteilung des Medikamentengemisches wurde Iohexol (Accupaque 240, GE Healthcare, Munich, Germany) verwendet, als Kortikoid wurden in Abhängigkeit vom Patientengewicht, Verträglichkeit sowie Vorerkrankungen 10–20 mg Triamcinolon injiziert (Volon A, (2 ×) 10 mg, Dermapharm AG, Gruenwald, Germany). Nach der Durchführung der Intervention wurden die Patienten gebeten, jeweils eine Stunde im Institut zu verweilen. Die anschließende Verabschiedung erfolgte vom durchführenden Arzt, um sicherzustellen, dass keine Nebenwirkungen aufgetreten sind. Unmittelbar nach der Therapie und im Abstand von 1 bis 14 Tagen (Durchschnitt: 8 Tage) wurde erneut der Schmerzscore mittels VAS bestimmt.

Die statistische Analyse wurde mittels SPSS V27 (IBM Armonk, USA) als t‑Test für unabhängige Stichproben durchgeführt. Die Autoren definierten *p* ≤ 0,05 als signifikant.

## Ergebnisse

Die Untersuchung umfasste 950 Interventionen bei 239 eingeschlossen Patienten. Im betrachteten Kollektiv befanden sich 136 (57 %) weibliche und 103 (43 %) männliche Patienten mit einem Durchschnittsalter von 57,6 Jahren. Bei 171 Patienten wurden insgesamt 686 PRT-Behandlungen durchgeführt, bei 68 Patienten insgesamt 264 Facetteninfiltrationen. Bei 40 Patienten wurde die PRT beidseitig durchgeführt, bei 131 Patienten einseitig, in der Gruppe der Patienten mit Facetteninfiltration wurde bei 41 Patienten eine beidseitige Facetteninfiltration vorgenommen, bei 27 eine einseitige.

Bei 71 % aller behandelten Patienten zeigte sich eine gute Beschwerdebesserung unter Therapie. Bei 29 % zeigte sich keine signifikante Besserung. Bei 74 % der Patienten in der PRT-Gruppe zeigte sich eine gute Beschwerdebesserung und entsprechend bei 60 % der Patienten in der FAC-Gruppe. Die durchschnittliche Schmerzreduktion in der Gruppe der PRT-Patienten betrug 41,3 %, in der Gruppe der FAC-Patienten 34,7 %.

Die Patienten erhielten 2 bis 6 CT-gesteuerte Interventionen an der Wirbelsäule, hierbei lag der Gesamtdurchschnitt bei 4,0 Untersuchungen. PRT-Patienten erhielten im Durchschnitt 4,1 Interventionen, FAC-Patienten 3,8 Intervention, wobei mit zunehmendem Grad der Arthrose die durchschnittliche Anzahl der durchgeführten Interventionen anstieg. Bei einer Spondylarthrose Grad 1 wurden im Mittel 3,1 Facettengelenkinfiltrationen durchgeführt, bei einer Spondylarthrose Grad 2 im Mittel 3,9 Interventionen, bei einer Spondylarthrose Grad 3 im Mittel 4,4 Interventionen. Der Abstand der Interventionen betrug 1 bis 78 Tage (Gesamtdurchschnitt: 8,5 Tage).

Von den insgesamt 950 Interventionen war die häufigste Lokalisation das Segment L5/S1 mit 306 Interventionen (220 PRT, 86 FAC), die zweithäufigste das Segment L4/L5 mit 299 Interventionen (209 PRT und 90 FAC) und die dritthäufigste das Segment C5/C6 mit 125 Interventionen (98 PRT, 27 FAC; Tab. 2 und 3). Die Höhe der Intervention zeigte sowohl bei der PRT wie auch bei der FAC keinen signifikanten Einfluss auf das Outcome.

**Table 2:** 

	Frauen	Männer	Gesamt
C3/4	5	0	5
C4/5	3	0	3
C5/6	61	37	98
C6/7	43	23	66
L1/2	4	0	4
L2/3	6	18	24
L3/4	34	23	57
L4/5	124	85	209
L5/S1	104	116	220
HWS	112	60	172
LWS	272	242	514
LWS + HWS	384 (56 %)	302 (44 %)	686

Anzahl der insgesamt durchgeführten PRT Interventionen mit Angabe des Segments bzw. der Höhe auf der die Intervention durchgeführt wurde

*HWS* Halswirbelsäule, *LWS* Lendenwirbelsäule, *PRT* periradikuläre Therapien

**Table 3:** 

	Frauen	Männer	Alle
C4/5	0	11	11
C5/6	17	10	27
C6/7	0	12	12
L1/2	5	0	5
L2/3	14	4	18
L3/4	10	5	15
L4/5	55	35	90
L5/S1	62	24	86
HWS	17	33	50
LWS	146	68	214
HWS + LWS	163	101	264

Anzahl der insgesamt durchgeführten FAC Interventionen mit Angabe des Segments bzw. der Höhe auf der die Intervention durchgeführt wurde

*HWS* Halswirbelsäule, *LWS* Lendenwirbelsäule, *FAC* Facettengelenkinfiltrationen

Bei den PRT-Patienten betrug die durchschnittliche Schmerzreduktion pro einzelner Intervention 0,76. Im Gesamtkollektiv der FAC-Patienten betrug die Schmerzreduktion pro Intervention durchschnittlich 0,66. Bezogen auf den Behandlungserfolg im Gesamtkollektiv zeigte die PRT gegenüber der Facettengelenkinfiltration ein signifikant besseres Ergebnis.

Das Therapieansprechen der Patienten mit Facettengelenkinfiltrationen unterscheidet sich in Abhängigkeit vom Arthrosegrad. Die durchschnittliche Schmerzreduktion pro Intervention nahm mit zunehmendem Arthrosegrad ab. Die durchschnittliche Schmerzreduktion pro Intervention lag bei einer Spondylarthrose Grad 1 bei 0,75. Verglichen mit den PRT-Patienten ergibt sich hier kein signifikanter Unterschied im Therapieansprechen.

Bei einer Spondylarthrose Grad 2 lag die durchschnittliche Schmerzreduktion pro Intervention bei 0,65, bei einer Spondylarthrose Grad 3 bei 0,61. Diese beiden Gruppen zeigen einen statistisch signifikanten Unterschied der durchschnittlichen Schmerzreduktion pro Intervention im Vergleich zur PRT-Gruppe (durchschnittliche Reduktion des Schmerzscores: 0,76).

**Figure 3:**
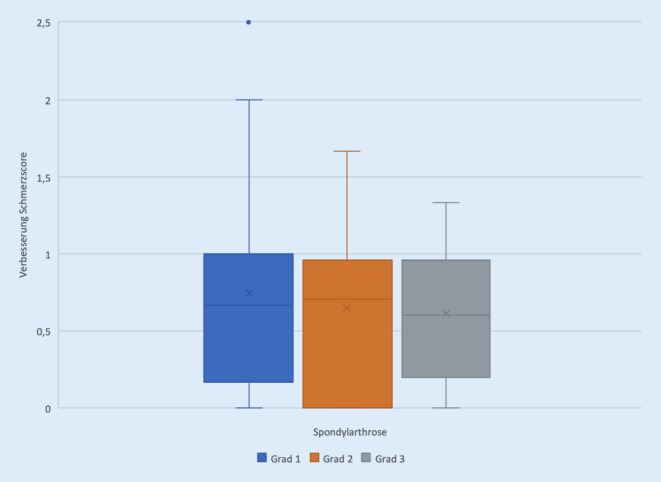

Betrachtet man für die Gesamtgruppen die durchschnittlich über den Behandlungszeitraum erzielte Reduktion des Schmerzscores, so zeigt hier die PRT-Gruppe mit einer Verbesserung um 3,1 das beste Outcome, gefolgt von der FAC-Gruppe mit dem höchsten Arthrosegrad mit einer Verbesserung um 2,5. Trotz nominell niedriger Schmerzreduktion pro Sitzung wird in dieser Gruppe über eine entsprechende Mehranzahl an Interventionen eine gute Schmerzreduktion erreicht (Abb. 3 und 4).

**Figure 4:**
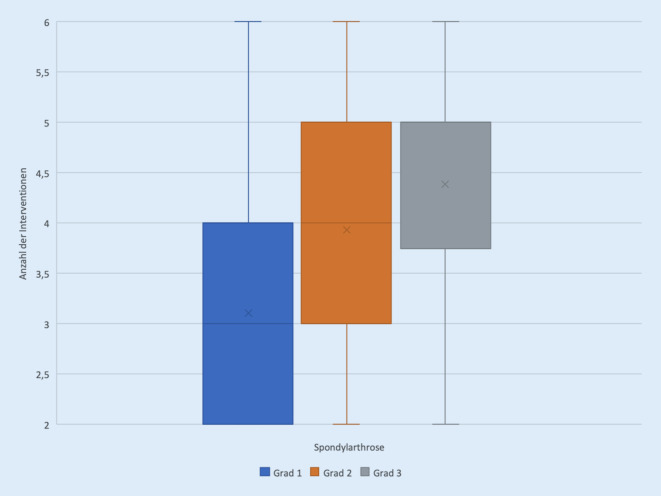

**Figure 5:**
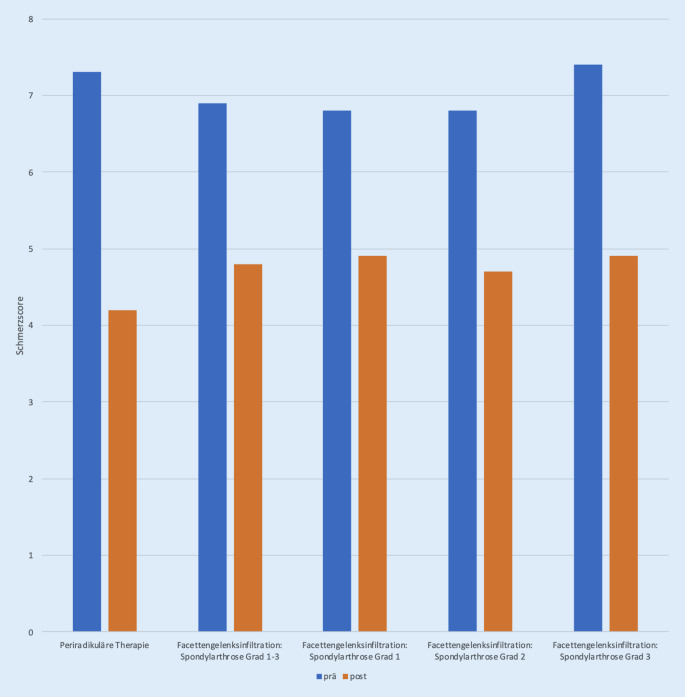

In der PRT-Gruppe betrug der Schmerzscore vor Intervention 7,3 (3–10), der Schmerzscore nach Intervention 4,2 (1–9). Im Gesamtkollektiv der FAC-Patienten betrug der Schmerzscore vor Intervention 6,9 (3–10) der Schmerzscore nach Intervention 4,8 (0–9). Bei den Patienten mit einer Facettengelenkinfiltration und einer Spondylarthrose Grad 1 betrug der Schmerzscore vor Intervention im Mittel 6,8 (3–9) auf der VAS, der Schmerzscore nach Intervention 4,9 (0–9). Bei den Patienten mit einer Facettengelenkinfiltration und einer Spondylarthrose Grad 2 betrug der Schmerzscore vor Intervention im Mittel 6,8 (4–10) auf der VAS, der Schmerzscore nach Intervention 4,7 (2–8). Bei den Patienten mit einer Facettengelenkinfiltration und einer Spondylarthrose Grad 3 betrug der Schmerzscore vor Intervention im Mittel 7,4 (4–10) auf der VAS, der Schmerzscore nach Intervention 4,9 (2–9; Abb. 5).

Die Nachevaluation konnte bei nur 37 % der Patienten telefonisch oder persönlich durchgeführt werden. Die Befragung hatte eine deutliche zeitliche Varianz zwischen 4 und 51 Wochen. Beide Patientengruppen berichten im Verlauf eine weitere Verbesserung mit einem durchschnittlichen Schmerzscore von 4,0 (0–8) für die PRT-Patienten und von 4,5 (0–8) für die FAC-Patienten.

## Diskussion

Diese Untersuchung zeigt eine Wirksamkeit sowohl der PRT wie auch der Facettentherapie im Hinblick auf die Verbesserung des Schmerzzustands des Patienten. In unserem Patientengut zeigten 74 % der PRT-Patienten und 60 % der FAC-Patienten eine gute Beschwerdebesserung. Diese Ergebnisse sind vergleichbar zur Literatur, auch hier wird eine gute Wirksamkeit der CT-gesteuerten Interventionen berichtet [32, 34, 37]. Wichtig für das Outcome scheint eine gute Patientenselektion mit klinisch plausibler Eruierung der Schmerzursache und konkordantem Bildbefund zu sein, da viele degenerative Wirbelsäulenveränderungen im Alter auftreten können, aber nicht zwingend zu Schmerzen führen müssen [13, 20]. Das in die Untersuchung eingeschlossene Patientengut war entsprechend vorselektiert, und es wurde bewusst auf einen eindeutigen Zusammenhang zwischen präsentierter Klinik und bildmorphologischem Befund Wert gelegt, um zu untersuchen, ob eine zielgerichtete Wirkung der Therapie eintritt. Ein insgesamt gutes Therapieansprechen sowohl im kurz- als auch mittelfristigen Verlauf bestätigten mehrere Untersuchungen, wobei gute Erfolge der periradikulären Therapie bei ca. 60–90 % der Patienten bezogen auf die Schmerzreduktion als auch funktionelle Aspekte beschrieben werden [29, 32, 33]. Eine größere vergleichende Untersuchung zeigt ebenfalls eine insgesamt gute Wirksamkeit der Therapie. Im Vergleich der verschiedenen Therapieformen ergeben sich auch in dieser vergleichenden Untersuchung deutliche Hinweise, dass eine gut auf die Klinik des Patienten angepasste Therapieform wichtiger ist als die technische Durchführung bzw. die verwendete Dosierung [7].

Die Spondylarthrose ist in Teilen eine regelhaft mit dem Alter vorkommende Erscheinung, die aber bei einer entsprechend starken Ausprägung auf zweierlei Weise zu Beschwerden führen kann. Zum einen kann die Arthrose selbst, insbesondere im Rahmen einer akuten Exazerbation mit lokaler entzündlicher Reizung der Facettengelenke oder auch im Rahmen eines chronisch-entzündlichen Prozesses zu Schmerz führen, zum anderen kann auch im Rahmen der Hypertrophie das Neuroforamen knöchern eingeengt und so über die knöcherne Enge des Neuroforamens eine Schmerzsymptomatik ausgelöst werden [12]. Das Facettengelenk ist ein anatomisch wichtiges und in seiner Gesamtauslegung im Rahmen der Wirbelsäulenmechanik ein komplexes Bewegungselement, das primär nur eine kleine segmentale Beweglichkeit erlaubt, im Rahmen eines Bewegungssegements aber im Zusammenspiel mit den benachbarten Wirbelgelenken entscheidend zur schmerzfreien Bewegung beiträgt. Jede Veränderung, jede Verletzung und letztlich auch jede Degeneration kann zu einer Veränderung der Bewegungsabläufe führen und direkt oder indirekt Schmerz auslösen und/oder verstärken [19]. Die Analyse der beteiligten Biomechanik sowie der Schmerzentstehung und Schmerztransformation sind hier noch Gegenstand der Forschung. Unsere Daten legen nahe, dass die Ausprägung der ursächlichen Pathologie einen Einfluss auf das Outcome hat. Die Patienten zeigen in unserer Untersuchung ein schlechteres Therapieansprechen pro einzelner Intervention bei steigendem Arthrosegrad. Diese Ergebnisse werden nur in Teilen von einer Arbeit an einem deutlich kleineren Patientenkollektiv von 50 Patienten von Kwak gestützt. In dieser Arbeit wurde die Facettengelenkarthrose ebenfalls in 3 Stufen eingeteilt und die Patienten einer jeweils gleich ausgelegten Facettengelenktherapie mit Glukokortikoid-Applikation zugeführt. In dieser Arbeit zeigt sich zwar konkordant zu unseren Ergebnissen generell eine Beschwerdebesserung in Form einer Reduktion des Schmerzscores, eine signifikante Abhängigkeit vom Arthrosegrad konnte aber nicht nachgewiesen werden. In den veröffentlichen Daten dieser Untersuchung zeigt sich aber übereinstimmend zu unseren Ergebnissen eine Abnahme des Erfolgs mit zunehmendem Grad der Facettengelenkarthrose [24]. Aufgrund der Pathoanatomie ist zu diskutieren, ob eine fortgeschrittene Facettengelenkarthrose aufgrund der Hypertrophie nicht auch in der Regel über die entstehende knöcherne neuroforaminale Enge zu einer begleitenden Nervenwurzelaffektion führt, so dass eine alleinige Facettengelenktherapie nicht den optimalen Behandlungserfolg gewährleisten kann. Der möglicherweise durch die knöcherne neurofoaminale Enge affektierte Nerv könnte ggf. von einer ergänzenden transforaminalen Therapie profitieren. So ist zu diskutieren, ob trotz eindeutiger Klinik für eine Spondylarthrose eine kombinierte Behandlung zu einem besseren Ergebnis geführt hätte [23].

Neu im Vergleich zu vielen radiologischen Studien ist in unserer Untersuchung der Vergleich zwischen PRT- und Facettengelenktherapie. Aus Sicht der Autoren ist dieser Vergleich sinnvoll, da der behandelnde Radiologe entscheiden muss, welche Behandlung erfolgversprechender erscheint, da gerade bei Patienten in fortgeschrittenem Alter häufig sowohl eine fortgeschrittene Spondylarthrose als auch eine Bandscheibenschädigung vorliegen kann. Hier sollten die Klinik und der Befund – im Idealfall um eine sorgfältige klinische/neurologische Untersuchung ergänzt – die sinnvollste und erfolgversprechendste Methode für den Patienten eruieren und entsprechend durchführen. In der Literatur wird bis zum jetzigen Zeitpunkt sehr kontrovers diskutiert, wie der spezifische Rückenschmerz entsteht und ob er sich überhaupt nur einer Ursache zuordnen lässt [38].

Die Schmerzwahrnehmung und Schmerzverarbeitung stellen ein multifaktorielles Geschehen dar, daher ist zu bedenken, dass in unserer Arbeit lediglich die Wirkung der mikroinvasiven CT-gesteuerten Therapie untersucht worden ist [3, 32]. Da die Kombination verschiedener Therapieelemente häufig auch zu einem verbesserten Therapie-Outcome und/oder zu einer Konsolidierung des Erfolgs im Langzeit-Outcome führt, ist zu diskutieren, wie die CT-gesteuerte Schmerztherapie sinnvoll in ein multimodales Konzept eingefügt werden könnte [14, 28].

Die Facettentherapie wird seit vielen Jahren in der Literatur kontrovers diskutiert [10]. Das Outcome zeigt in verschiedenen Studien eine breite Streuung, zudem wird neben der Injektion von Kortikoiden auch häufig die physikalische Denervation untersucht, auch hier mit unterschiedlichen Ergebnissen [6, 15, 31]. In der Literatur ist festzustellen, dass auch bei der Facettentherapie – sei es mittels intraartikulärer Kortikoid-Injektion oder mittels physikalischer Denervation – sehr genau die Klinik des Patienten beachtet werden muss [10, 15].

Bezüglich der Geschlechtsverteilung waren in der Gesamtgruppe Frauen mit 55 % etwas häufiger vertreten als Männer. Diese Tendenz zeigt sich auch in anderen Studien, so waren in einer vergleichenden Veröffentlichung aus 2009 52–57 % aller Schmerzpatienten mit unterem Rückenschmerz weiblich [26]. Konkordant zu dieser Untersuchung ist auch im eigenen Patientenkollektiv die häufigste Schmerzlokalisation im Bereich der Lendenwirbelsäule, insbesondere die Segmente LW4/5 und LW5/SW1 waren am häufigsten betroffen und wurden am häufigsten behandelt [26]. Unsere Untersuchung zeigt keine signifikanten Wirkungsunterschiede sowohl der FAC wie auch der PRT bzgl. des Geschlechts der Patienten.

Am postinterventionellen Follow-up nahmen nur 37 % der Patienten teil. Der Effekt relativ geringer Teilnehmerquoten im Rahmen von Nachuntersuchungen ist allgemein bekannt [30]. Es werden verschiedene mögliche Einflussfaktoren diskutiert – sowohl beim Patienten als auch bei der Art der Nachbefragung und bei der Art der Einladung zu einer Nachbefragung [27, 30]. Von daher wurde eine spätere stichprobenartige telefonische Nachbefragung bei einigen Patienten, die am Follow-up nicht teilgenommen hatten, durchgeführt. Diese ergab verschiedene Gründe für die Nichtteilnahme: Vergessen der Nachbefragung, gutes Behandlungsergebnis, von daher aus Sicht des Patienten keine Notwendigkeit zur nochmaligen Kontaktaufnahme, keine Zeit.

### Limitationen

In der vorliegenden Untersuchung wurden lediglich die Methoden der PRT- und FAC-Therapie untersucht, eine mögliche Einflussnahme durch den behandelnden Arzt oder weitere psychologische Faktoren wurde in der vorliegenden Arbeit nicht berücksichtigt. Die Gruppe der FAC-Patienten war deutlich kleiner, daher ist hier die Sicherheit der Aussagen geringer als in der PRT-Gruppe. Die Quote der im Langzeitverlauf nachbefragten Patienten ist mit 37 % sehr gering ausgefallen, zusätzlich zeigt der Zeitraum der Nachbefragung eine große zeitliche Varianz. Insofern kann zum Langzeit-Outcome keine valide Aussage getroffen werden. Auch aufeinander aufbauende Therapieoptionen wurden in der vorliegenden Untersuchung nicht berücksichtigt.

## Fazit für die Praxis

- Die CT-gesteuerte periradikuläre Therapie und die CT-gesteuerte Facettengelenktherapie führen beide zu einer guten Reduktion der Beschwerdesymptomatik.
- Die PRT zeigt durchschnittlich eine bessere Schmerzreduktion gegenüber der FAC.
- Es zeigt sich eine negative Korrelation zwischen Grad der Degeneration und Wirksamkeit der Therapie pro Intervention.
- Durch eine Erhöhung der Anzahl an Interventionen kann im Vergleich auch bei massiven degenerativen Veränderungen der Facettengelenke eine gute therapeutische Wirkung erzielt werden.
